# Physical Fitness and External Training Load Represent Distinct Dimensions of Performance in Female Football Players During the Pre-Season

**DOI:** 10.3390/sports14050206

**Published:** 2026-05-18

**Authors:** Artur Avelino Birk Preissler, Filipe Manuel Clemente, Ewerton Luiz Bourscheid da Rocha, Rui Miguel Silva, Ana Filipa Silva, Jocelito Bijoldo Martins, Pedro Schons

**Affiliations:** 1 Faculdade SOGIPA, Porto Alegre 90240-485, RS, Brazilpedroschons@hotmail.com (P.S.); 2 School of Physical Education, Physiotherapy and Dance, Federal University of Rio Grande do Sul, Porto Alegre 90690-200, RS, Brazil; 3 Department of Biomechanics and Sport Engineering, Gdansk University of Physical Education and Sport, 80-336 Gdańsk, Poland; filipe.clemente5@gmail.com; 4 Applied Research Institute (i2A), Polytechnic University of Coimbra, 3045-093 Coimbra, Portugal; 5 The Sport Physical Activity and Health Research & Innovation Center, 3045-093 Coimbra, Portugal; 6 Escola Superior de Desporto e Lazer, Instituto Politécnico de Viana do Castelo, 4960-320 Viana do Castelo, Portugal; 7 The Sport Physical Activity and Health Research & Innovation Center, 4960-320 Viana do Castelo, Portugal; 8 Faculty of Sport Sciences and Physical Education, University of Coimbra, 3040-256 Coimbra, Portugal

**Keywords:** female football, external training load, physical fitness, preseason, principal component analysis

## Abstract

Monitoring performance in football often combines physical testing and GPS-derived external-load measures, although their relationships remain unclear. This study examined the relationships between physical-test outcomes and GPS-derived external-load variables during the pre-season in professional female football players and whether these measures appear to capture distinct dimensions of performance. This observational study monitored 24 outfield players from a Brazilian Women’s First Division team during a 6-week pre-season. Players performed the countermovement jump, 10 m and 30 m sprints, change-of-direction test, and 30–15 intermittent fitness test while external load was recorded across field sessions. Associations were examined using Pearson’s or Spearman’s correlations, and principal component analysis (PCA) was applied. Significant correlations were more frequent within than between domains. Total distance correlated with accelerations (ρ = 0.740, *p* < 0.001), decelerations (ρ = 0.684, *p* < 0.001), Z3 distance (ρ = 0.595, *p* = 0.003), and Z4 distance (ρ = 0.584, *p* = 0.003), while sprint count correlated with sprint distance (r = 0.950, *p* < 0.001). Estimated VO_2_max correlated positively with CMJ (r = 0.533, *p* = 0.007) and negatively with 10 m (r = −0.445, *p* = 0.029) and 30 m sprint times (r = −0.476, *p* = 0.019). PCA identified two components explaining 61.4% of the total variance: external load (40.6%) and physical performance (20.8%). These findings indicate that both approaches capture distinct and complementary aspects of performance.

## 1. Introduction

Women’s football requires players to sustain substantial total locomotor volume while repeatedly performing high-intensity running and sprint actions that are sensitive to competitive level and playing position [1,2,3]. The movement patterns of women’s football also show marked contextual and positional variability, which makes performance monitoring central to training design and interpretation [3,4]. Despite rapid growth in the women’s game, the monitoring literature remains less mature than in men’s football, and recent expert reviews still identify major evidence gaps in female-specific readiness and performance surveillance [5,6].

Global positioning system (GPS)-derived external-load measures are now widely used to quantify the volume and intensity of football training through variables such as total distance, speed-zone distance, sprint exposure, and acceleration or deceleration counts [7]. However, the interpretation of those measures is affected by device characteristics, processing choices, and the lack of uniform thresholds for locomotor categories, which complicates both comparison across studies and direct inference about physical capacity [7,8]. For that reason, field-based physical testing remains recommended in football to characterize complementary qualities such as lower-limb power, linear speed, change-of-direction ability, and intermittent-aerobic fitness [9,10]. Among aerobic field tests, the 30–15 Intermittent Fitness Test has shown validity and reliability in female soccer players and appears particularly useful for tracking changes relevant to intermittent performance [10,11,12].

The current female-specific literature suggests that training-load monitoring can inform performance management, but the evidence base is still dominated by observational single-team studies and remains methodologically heterogeneous [6,13]. Available evidence indicates that accumulated external load during pre-season may be associated with improvements in intermittent-exercise capacity, and cohort studies in adult women’s soccer have linked better fitness status with superior match-running outputs, especially at higher intensities [1,13,14,15]. At the same time, broader team-sport evidence indicates that external-load measures alone often show limited associations with physical-performance adaptations, whereas some internal-load metrics display more consistent relationships with endurance-related change [16,17].

In some applied contexts, there has been a tendency to prioritize training-load monitoring due to practical constraints, such as congested competition schedules and reduced preparation time, under the assumption that training demands may sufficiently reflect players’ physical status. Accordingly, it remains uncertain whether routine GPS-derived external load can serve as a proxy for standard physical testing in professional female football or whether both approaches provide distinct and complementary information [6,9,16]. Although physical fitness and training-load variables have been investigated separately in female football, evidence directly integrating both approaches during the pre-season remains limited, particularly in professional players. Multivariate studies in football monitoring have shown that training-load variables frequently resolve into separate latent components and that the structure of those relationships may differ by season phase, particularly during the pre-season period [18,19,20]. Yet direct evidence integrating a pre-season physical-test battery with routine external training-load profiling in professional female football remains scarce [6,13].

Therefore, the present study examined the relationships between countermovement jump, sprint, change of direction, and estimated VO_2_max derived from the 30–15 Intermittent Fitness Test and GPS-derived external-load variables across a six-week pre-season in professional female outfield football players. We also used principal component analysis to determine whether these measures mapped onto common or distinct performance dimensions. We hypothesized that physical tests and external-load measures would present limited associations and would cluster into distinct components, reflecting complementary rather than interchangeable dimensions of performance.

## 2. Materials and Methods

### 2.1. Participants

Participants were outfield players from a professional female football team competing in the Brazilian Women’s First Division. The study was designed as an observational, descriptive, and quantitative investigation conducted over six weeks during the pre-season period. Participants were recruited using a convenience sampling approach, based on their availability to complete the physical testing battery and to be monitored throughout the training period. The sample size reflected the total number of eligible outfield players available within the professional team during the monitoring period. The sample consisted of 24 players, including midfielders (*n* = 8), forwards (*n* = 7), center-backs (*n* = 6), and full-backs (*n* = 3). Goalkeepers were excluded due to the specific physical demands of their position. Additionally, players who did not complete the monitoring period or who sustained injuries, as determined by the club’s medical staff, were excluded from the analysis. All assessments were conducted within the team’s regular training environment, ensuring minimal disruption to routine and enhancing the ecological validity of the data. The study was conducted in accordance with the principles of the Declaration of Helsinki. All participants provided written informed consent prior to participation. Ethical approval was granted by the Research Ethics Committee of the Pontifical Catholic University of Rio Grande do Sul (CEP-PUCRS) (Approval number: 6.997.111; date of approval: 2 September 2024).

### 2.2. Design and Setting

Data were collected from a professional female football team competing in the Brazilian Women’s First Division during the pre-season period. This study adopted an observational design conducted in an applied training setting. Anthropometric data (age, height, and body mass) and physical performance measures were obtained, including countermovement jump (CMJ), linear sprint performance over 10 m and 30 m, change-of-direction ability (COD), and maximal oxygen uptake estimated from the 30–15 Intermittent Fitness Test (VO_2_max). All physical assessments were performed during a single testing session in the second week of the pre-season, following protocols previously validated for football settings. These assessments were part of the team’s regular monitoring routine and were used to characterize the players’ physical performance profile. The testing session was performed under consistent conditions, typically at the same time of day within the team’s training schedule. Participants were instructed to avoid strenuous physical activity prior to testing. Before the assessments, participants performed a standardized warm-up consisting of dynamic exercises commonly used in their training routines. External training load was monitored using GPS devices across all field training sessions throughout the six-week pre-season. The use of GPS devices was also part of the team’s regular monitoring practices, ensuring that data collection did not interfere with the training process. For each participant, GPS-derived variables were summarized using the individual median across the monitoring period in order to reduce the influence of occasional extreme observations and to represent the players’ typical external-load profile during the pre-season.

### 2.3. Procedures

Female football players underwent assessments of anthropometric characteristics and physical performance. The test battery included: (i) body mass and stature; (ii) countermovement jump (CMJ); (iii) linear sprint performance over 10 m and 30 m; (iv) change-of-direction ability (COD); and (v) intermittent aerobic performance assessed through the 30–15 Intermittent Fitness Test, from which maximal oxygen uptake (VO_2_max) was estimated. Participants were instructed to maintain their usual dietary habits and to avoid strenuous physical activity prior to testing. All assessments were conducted following standardized procedures, with prior familiarization and verbal instructions provided by the evaluators.

#### 2.3.1. Anthropometric Assessment

Anthropometric variables included height and body mass. Height was measured using a wall-mounted stadiometer with a precision of 1 mm. Participants were assessed barefoot in a standing position, with heels together, body upright, and head positioned in the Frankfurt plane. Body mass was measured using a digital scale with a precision of 0.1 kg. Participants were assessed barefoot and wearing light clothing, remaining still until the measurement stabilized. Values were recorded in kilograms (kg).

#### 2.3.2. Vertical Jump Test

Lower-limb power was assessed using the countermovement jump (CMJ), performed on a contact platform (Jump System, Cefise, Nova Odessa, Brazil), connected to a portable computer for data acquisition. Participants started from an upright standing position with their hands on their hips. Following a verbal command, they performed a rapid downward movement (approximately 90° knee flexion), immediately followed by a maximal concentric extension of the lower limbs to execute the jump. Two attempts were performed, with a 30 s interval between them, and the highest value was retained for analysis and expressed in centimeters [21,22]. Jump height was determined based on flight time recorded by the system and calculated using the equation h = g × t^2^/8, where h represents jump height, g is the acceleration due to gravity, and t is the flight time.

#### 2.3.3. Linear Sprint Tests

Linear sprint tests over 10 m and 30 m were performed to assess acceleration and running speed, respectively. Testing was conducted on-field using an electronic timing system with photocells (CEFISE, São Paulo, Brazil), with a precision of 1 ms. Photocells were positioned at a height of 100 cm. Participants started from a standardized position, with the front foot placed 0.30 m behind the first timing gate and the trunk slightly inclined forward. Following an auditory signal, participants sprinted maximally over the prescribed distances. For the 10 m test, timing gates were positioned at the start line and at 10 m. For the 30 m test, gates were placed at the start and at 30 m. In both tests, a cone was placed 5 m beyond the finish line to prevent premature deceleration before crossing the final gate [23,24]. Each participant performed two trials for each distance, with a 3 min passive recovery between attempts. The fastest time recorded for each distance was used for analysis.

#### 2.3.4. Change-of-Direction Test

Change-of-direction ability was assessed using the 20 m Zig-zag test, consisting of four 5 m sections marked by cones positioned at 100° angles, which participants were required to navigate externally. The objective was to complete the course in the shortest possible time. Testing was conducted using an electronic timing system with photocells (CEFISE, São Paulo, Brazil), positioned at a height of 100 cm at the start and end of the course. Participants started from a position 0.30 m behind the first timing gate. A cone was placed 5 m beyond the finish line to prevent premature deceleration [23,24]. Each participant performed two trials, with a 3 min passive recovery between attempts. The fastest time recorded was used for analysis and expressed in seconds.

#### 2.3.5. 30–15 Intermittent Fitness Test

Intermittent aerobic capacity was assessed using the 30–15 Intermittent Fitness Test (30–15 IFT), which consists of repeated 30 s shuttle runs interspersed with 15 s of passive recovery. The test started at a speed of 8 km·h^−1^, with increments of 0.5 km·h^−1^ at each stage, with running speed controlled by audio signals. Participants were required to reach predefined zones at each end of the course, and the test was terminated when the participant failed to reach the target zone on three consecutive occasions. The primary variable obtained was the final velocity reached at the last completed stage (VIFT), expressed in km·h^−1^. Estimated maximal oxygen uptake (VO_2_max), derived from VIFT, was used for subsequent analyses and interpretation of aerobic performance. Maximal oxygen uptake (VO_2_max) was estimated from VIFT using the equation proposed by Buchheit [25,26].

#### 2.3.6. External Load Monitoring Using GPS

External load variables were monitored across all field-based training sessions during the six-week pre-season period using global positioning system devices (GPS; Catapult Sports, Melbourne, Australia) operating at a sampling frequency of 10 Hz. The units were positioned on the upper interscapular region of the participants using manufacturer-provided vests to ensure stability during movement. The devices were activated approximately 10 min before each session to allow signal stabilization. Data were downloaded and processed using the Catapult OpenField software 3.12.0 and included variables related to training volume and intensity. The analyzed metrics comprised total distance covered (m), peak velocity (km·h^−1^), number of sprints (defined as efforts > 23 km·h^−1^), sprint distance (m), and the number of accelerations and decelerations (defined as changes in velocity ≥ 2 m·s^−2^ and ≤−2 m·s^−2^, respectively). Distance covered was categorized into predefined velocity zones according to reference thresholds adopted in team sport monitoring, as follows: Z1 (≤7 km·h^−1^), Z2 (7–13 km·h^−1^), Z3 (13–19 km·h^−1^), Z4 (19–23 km·h^−1^), and Z5 (>23 km·h^−1^). Absolute thresholds were adopted because they were part of the team’s routine monitoring practices and allowed comparisons with previous football studies using similar GPS metrics. For each participant, all variables were summarized using the individual median across the six-week monitoring period, representing the typical external load profile during the pre-season [27].

### 2.4. Statistical Analysis

Descriptive statistics were expressed as mean ± standard deviation. Data normality was assessed using the Shapiro–Wilk test. Data distribution and potential outliers were visually inspected using boxplots prior to inferential analyses, and no extreme outliers requiring removal were identified. Bivariate correlations were performed using Pearson’s correlation coefficient when both variables in the pair were normally distributed and Spearman’s rank correlation coefficient when at least one variable in the pair did not meet the normality assumption. Among the original variables, those that did not meet the normality assumption were total distance, accelerations, distance in zone 2, and distance in zone 5. The magnitude of the correlations was qualitatively interpreted according to the criteria proposed by Hopkins (2000), classified as: trivial (r = 0), small (0 < r ≤ 0.30), moderate (0.30 < r ≤ 0.60), large (0.60 < r ≤ 0.90), very large (0.90 < r < 1.00), and nearly perfect (r = 1.00) [28]. To investigate the structure of the variables, a principal component analysis (PCA) with varimax rotation was performed. Sampling adequacy was assessed using Bartlett’s test of sphericity and the Kaiser–Meyer–Olkin (KMO) index. Component retention was based on parallel analysis, retaining components with eigenvalues exceeding those obtained from random data. Factor loadings ≥0.40 were considered for interpretation [29,30]. The level of significance was set at α < 0.05 (two-tailed). All analyses were performed using Jamovi 2.6.26.

## 3. Results

Descriptive characteristics of the participants are presented in Table 1. The participants had a mean age of 27.03 ± 4.59 years, body mass of 64.86 ± 6.82 kg, and height of 167.79 ± 6.06 cm. Regarding physical performance, mean values were 32.30 ± 4.65 cm for CMJ, 1.88 ± 0.08 s for the 10 m sprint, 4.61 ± 0.18 s for the 30 m sprint, 5.49 ± 0.17 s for the change-of-direction test (COD), and 46.86 ± 2.06 mL·kg^−1^·min^−1^ for estimated VO_2_max derived from the 30–15 Intermittent Fitness Test. For external load variables, the mean total distance was 3470.42 ± 383.97 m, with 3.31 ± 2.15 sprints per session and a sprint distance of 106.09 ± 58.30 m. Mean maximum velocity was 23.43 ± 1.34 km·h^−1^, with 95.81 ± 19.68 accelerations and 93.60 ± 19.51 decelerations. Distances covered across the different intensity zones are also presented in Table 1.

Correlations between variables are presented in Table 2. Significant correlations were predominantly observed between variables of similar nature. Among external load variables, the strongest positive associations were observed between total distance and accelerations (ρ = 0.740; *p* < 0.001), sprint count and sprint distance (r = 0.950; *p* < 0.001), sprint distance and maximum velocity (r = 0.768; *p* < 0.001), and between variables related to higher intensity zones, particularly Z3 and Z4. For physical performance variables, estimated VO_2_max was positively correlated with CMJ (r = 0.533; *p* = 0.007) and negatively correlated with sprint times over 10 m (r = −0.445; *p* = 0.029) and 30 m (r = −0.476; *p* = 0.019). Positive correlations were also observed between sprint tests (10 m and 30 m: r = 0.710; *p* < 0.001) and between the 30 m sprint and COD (r = 0.531; *p* = 0.008). Regarding the principal components, PC1 was predominantly characterized by external load variables, whereas PC2 was predominantly characterized by physical performance variables, including CMJ, sprint performance, COD, and estimated VO_2_max. Overall, few significant correlations were observed between physical performance and external load variables.

**Figure 1:**
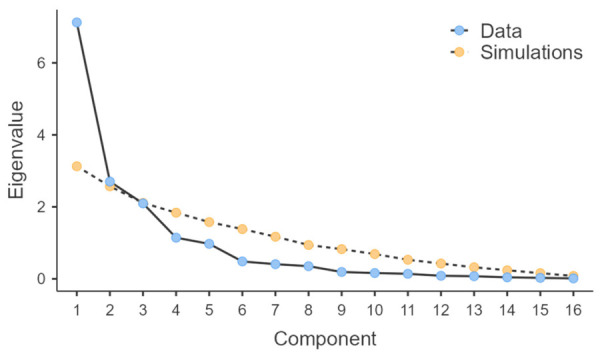
Scree plot from the parallel analysis used to determine the number of retained principal components. Observed eigenvalues from the study data are compared with simulated eigenvalues. Components were retained when the observed eigenvalue exceeded the corresponding simulated eigenvalue, supporting the two-component solution.

The adequacy of the data for principal component analysis was confirmed by Bartlett’s test of sphericity (χ^2^ = 349; df = 120; *p* < 0.001), indicating the presence of significant correlations among variables. The Kaiser–Meyer–Olkin index (KMO = 0.709) indicated adequate sampling for the analysis. Parallel analysis indicated the retention of two principal components, as illustrated in Figure 1. After varimax rotation, these components presented eigenvalues of 6.50 and 3.33, explaining 40.6% and 20.8% of the total variance, respectively, accounting for 61.4% of the total variance.

The factor loadings of the extracted components are presented in Table 3, and only loadings with a magnitude greater than 0.40 were considered for interpretation. Higher absolute loadings indicate a stronger association between the variable and the component. The first component was predominantly characterized by variables related to external load, including total distance, sprint-related metrics, maximum velocity, accelerations, decelerations, and distances covered across intensity zones. The second component was predominantly characterized by variables related to physical performance, particularly sprint performance, change of direction, estimated VO_2_max, and countermovement jump.

## 4. Discussion

This study found that pre-season physical-test results and GPS-derived external training-load variables were only weakly related in professional female football players. Stronger correlations were observed mainly among variables of the same type, and principal component analysis retained two components that largely separated external-load metrics from physical-performance tests. These findings suggest that the monitored training exposure and the players’ measured physical capacities represented complementary rather than interchangeable dimensions of performance during the pre-season.

The clearest interpretation of our results is that external training load does not appear to act as a direct surrogate for physical fitness status in this context. This reading is broadly consistent with a recent team-sport meta-analysis showing that external-load measures tend to display limited associations with physical-performance adaptations, whereas heart-rate-based internal-load metrics show more consistent relationships with endurance-related change [17]. A systematic review on the internal-to-external efficiency approach points in the same direction, namely that combining load domains may be informative for fitness surveillance, but that measure selection materially affects sensitivity and that evidence in female athletes remains limited [16]. Studies in football monitoring likewise suggest that load variables often resolve into more than one latent dimension, especially in the pre-season, when volume and intensity may be less tightly coupled than during competitive phases [18,19,20]. Our findings extend that logic to professional female football by showing that standardized physical testing and typical pre-season GPS exposure organize into separate performance domains.

Within the external-load domain, the strong relationships among sprint count, sprint distance, maximum velocity, and higher-speed zones were expected because these measures capture overlapping features of high-speed locomotor exposure [7,8]. The positive associations of total distance with accelerations, decelerations, and intermediate-to-high-speed distances similarly reflect the partial redundancy that is well recognized in GPS-derived monitoring outputs [7]. Female-specific microcycle studies have shown that external-load profiles vary meaningfully according to session day and playing position and that sprint-related measures may behave differently from broader volume indicators, which reinforces the need for multidimensional interpretation rather than reliance on a single summary variable [3,31]. The cross-loading of the highest-speed zone in our PCA is therefore plausible, because maximal-speed exposure is simultaneously shaped by training prescription and by the locomotor capacities that enable players to reach those thresholds [1,2,32].

Within the physical-test domain, the grouping of countermovement jump, sprint, change of direction, and estimated VO_2_max supports the view that a pre-season test battery can capture a coherent profile of neuromuscular and intermittent-aerobic capacity. This interpretation aligns with recent testing recommendations in soccer, which treat jump, linear speed, multidirectional speed, and intermittent aerobic assessments as complementary measures of distinct but interacting physical qualities [9,10]. The 30–15 Intermittent Fitness Test is especially relevant in this setting because it is valid and reliable in female soccer players and appears sensitive to soccer-specific changes in aerobic fitness [10,11,12]. Female-soccer studies have also reported that better fitness status is associated with superior match-running outputs, particularly for intermittent or high-intensity actions, although not all running measures are explained equally well by the same tests [1,15]. Our results refine the literature by suggesting that these capacities are expressed more clearly in standardized testing than in players’ median external-load exposure during the pre-season.

The limited correlations between domains should not be interpreted as evidence that fitness is unimportant for football performance. Rather, they suggest that the external load accumulated in training is strongly constrained by coaching objectives, task design, positional role, and season phase, all of which can decouple what a player is physically capable of from what she is actually asked to perform in training [13,19,31]. This is particularly plausible during the pre-season, when technical, tactical, and conditioning goals are pursued concurrently and when progression of training intensity may matter as much as or more than accumulated volume alone [13,33,34]. Similar findings have also been reported in other ecologically valid sport settings, where GNSS-derived external-output variables showed limited associations with physiological and perceptual internal-load indicators [35]. In methodological terms, our use of player-specific medians across six weeks was appropriate for characterizing typical exposure, but that approach may attenuate short-lived peaks and within-player dose–response patterns that become more visible in longitudinal adaptation studies [14,17,33]. In addition, the temporal separation between the physical-testing session and the six-week aggregation of external-load data may also have contributed to attenuating the observed associations between domains, particularly for variables that are sensitive to short-term fluctuations in fitness and training exposure.

This study should be interpreted in light of some limitations. The sample was drawn from a single professional team and was modest in size, which limits position-specific inference and reduces external validity. No formal a priori power analysis was conducted, as the sample size was determined by the number of eligible players available within the professional team during the monitoring period. Therefore, the correlation and PCA findings should be interpreted cautiously, particularly considering the applied single-team context, the modest sample size, and the number of variables analyzed. The observational design precludes causal interpretation. In addition, physical fitness was assessed during a single testing session, whereas external load was summarized across six weeks; this temporal mismatch may have reduced the sensitivity to detect stronger associations between physical fitness and external-load variables. Similarly, summarizing GPS-derived variables using individual median values provided a practical estimate of typical exposure but may have reduced sensitivity to intra-individual fluctuations, peak loads, and short-term dose–response dynamics. Although sampling adequacy indices supported the use of PCA, the identified component structure should be viewed as exploratory rather than definitive evidence of stable latent performance constructs. Furthermore, maximal oxygen uptake was estimated from the 30–15 test rather than measured directly, and the study did not integrate internal-load, wellness, menstrual-cycle, or match-play variables that could help explain inter-individual variation. Future research should use multicenter samples in women’s football, repeated fitness assessments, and integrated internal-external monitoring frameworks to investigate whether longitudinal changes in external load are associated with changes in distinct physical capacities across different phases of the season and according to positional demands.

From a practical perspective, these data suggest that pre-season monitoring should not be reduced to GPS-derived external-load dashboards alone. Standardized physical tests and routine external-load monitoring appear to inform different decisions, with the former describing the players’ neuromuscular, speed, and intermittent-aerobic capacities and the latter describing the volume and intensity actually imposed by training [9]. Practitioners may therefore benefit from retaining a lean but multidimensional monitoring system rather than assuming that one modality can replace the other [9]. Data-reduction approaches can still be useful for simplifying monitoring dashboards, provided that the retained variables continue to reflect both external-load and physical-performance constructs within the practical and contextual demands of the applied setting.

## 5. Conclusions

In professional female football players during the pre-season, standard physical tests and GPS-derived external training-load variables showed limited overlap and were organized predominantly into separate performance dimensions. External-load metrics clustered around the volume and intensity of the work imposed by training, whereas physical-test outcomes clustered around players’ physical capacities. These findings indicate that routine GPS monitoring should not be interpreted as a substitute for pre-season physical testing in this population. Instead, combining both approaches offers a more robust way to characterize player status and training exposure during the pre-season.

## Figures and Tables

**Table 1:** Descriptive characteristics, physical performance, and external load variables of professional female football players during the pre-season.

	Mean	SD	Lower CI (95%)	Upper CI (95%)
Age (y)	27.03	4.59	25.09	28.97
BM (kg)	64.86	6.82	61.98	67.74
H (cm)	167.79	6.06	165.23	170.35
CMJ (cm)	32.30	4.65	30.34	34.26
S10 (s)	1.88	0.08	1.85	1.91
S30 (s)	4.61	0.18	4.53	4.68
COD (s)	5.49	0.17	5.42	5.56
VO_2_max (mL·kg^−1^·min^−1^)	46.86	2.06	45.99	47.73
TD (m)	3470.42	383.97	3308.29	3632.56
SC (n)	3.31	2.15	2.41	4.22
SpD (m)	106.09	58.30	81.47	130.71
Vmax (km·h^−1^)	23.43	1.34	22.87	24.00
Acc (n)	95.81	19.68	87.50	104.12
Dec (n)	93.60	19.51	85.37	101.84
Z1 (m)	2080.75	248.33	1975.89	2185.61
Z2 (m)	975.96	176.48	901.44	1050.48
Z3 (m)	443.38	125.61	390.34	496.41
Z4 (m)	84.46	44.75	65.57	103.36
Z5 (m)	11.69	15.11	5.31	18.07

Abbreviations: SD = standard deviation; CI = confidence interval; BM = body mass; H = height; CMJ = countermovement jump; S10 = 10 m sprint; S30 = 30 m sprint; COD = change of direction; VO_2_max = maximal oxygen uptake (estimated from 30–15 IFT); TD = total distance; SC = sprint count; SpD = sprint distance; Vmax = maximum velocity; Acc = accelerations; Dec = decelerations; Z1–Z5 = velocity zones. External-load variables represent individual median values across the six-week pre-season monitoring period.

**Table 2:** Correlation matrix between physical performance and external load variables in professional female football players during the pre-season.

		CMJ	S10	S30	COD	VO_2_max	TD	SC	SpD	Vmax	Acc	Dec	Z1	Z2	Z3	Z4	Z5	PC1
S10	*r*	−0.333	—															
	*p*	0.112	—															
S30	*r*	−0.241	0.710	—														
	*p*	0.257	<0.001 *	—														
COD	*r*	−0.168	0.486	0.531	—													
	*p*	0.434	0.016 *	0.008 *	—													
VO_2_max	*r*	0.533	−0.445	−0.476	−0.365	—												
	*p*	0.007 *	0.029 *	0.019 *	0.079	—												
TD	ρ	0.150	−0.132	−0.299	−0.190	0.348	—											
	*p*	0.484	0.538	0.155	0.373	0.096	—											
SC	*r*	−0.088	−0.288	−0.259	−0.120	0.245	0.397	—										
	*p*	0.681	0.172	0.221	0.576	0.248	0.055	—										
SpD	*r*	−0.033	−0.334	−0.310	−0.121	0.253	0.477	0.950	—									
	*p*	0.880	0.111	0.141	0.572	0.233	0.020 *	<0.001 *	—									
Vmax	*r*	0.156	−0.390	−0.385	−0.174	0.336	0.494	0.704	0.768	—								
	*p*	0.467	0.059	0.063	0.416	0.108	0.015 *	<0.001 *	<0.001 *	—								
Acc	ρ	0.408	−0.237	−0.431	−0.044	0.355	0.740	0.257	0.359	0.558	—							
	*p*	0.048 *	0.266	0.035 *	0.839	0.088	<0.001 *	0.225	0.085	0.005 *	—							
Dec	*r*	0.179	−0.250	−0.225	0.010	0.120	0.684	0.334	0.414	0.722	0.779	—						
	*p*	0.404	0.239	0.289	0.962	0.575	<0.001 *	0.111	0.044	<0.001 *	<0.001 *	—						
Z1	*r*	0.077	0.159	−0.146	−0.284	0.169	0.593	0.057	0.125	0.294	0.331	0.379	—					
	*p*	0.720	0.458	0.496	0.178	0.431	0.003 *	0.793	0.560	0.164	0.115	0.068	—					
Z2	ρ	−0.249	−0.101	−0.013	0.135	−0.040	0.464	0.120	0.270	0.205	0.451	0.302	0.319	—				
	*p*	0.241	0.640	0.953	0.529	0.852	0.023 *	0.575	0.200	0.334	0.027 *	0.152	0.129	—				
Z3	*r*	−0.147	−0.241	−0.184	−0.113	0.126	0.595	0.774	0.873	0.787	0.428	0.594	0.294	0.311	—			
	*p*	0.494	0.256	0.388	0.600	0.557	0.003 *	<0.001 *	<0.001 *	<0.001 *	0.037 *	0.002 *	0.163	0.139	—			
Z4	*r*	0.027	−0.262	−0.251	−0.154	0.208	0.584	0.871	0.942	0.716	0.414	0.348	0.185	0.235	0.850	—		
	*p*	0.900	0.216	0.237	0.473	0.329	0.003 *	<0.001 *	<0.001 *	<0.001 *	0.044 *	0.095	0.386	0.268	<0.001 *	—		
Z5	ρ	0.328	−0.313	−0.531	−0.166	0.381	0.475	0.772	0.832	0.860	0.472	0.497	0.070	0.049	0.756	0.779	—	
	*p*	0.118	0.137	0.008 *	0.437	0.066	0.019 *	<0.001 *	<0.001 *	<0.001 *	0.020 *	0.014 *	0.747	0.821	<0.001 *	<0.001 *	—	
PC1	ρ	−0.035	−0.182	−0.248	0.081	0.173	0.730	0.760	0.843	0.738	0.621	0.580	0.287	0.497	0.874	0.850	0.698	—
	*p*	0.870	0.395	0.242	0.706	0.420	<0.001 *	<0.001 *	<0.001 *	<0.001 *	0.001 *	0.003 *	0.174	0.014 *	<0.001 *	<0.001 *	<0.001 *	—
PC2	*r*	0.537	−0.769	−0.753	−0.602	0.752	0.143	0.383	0.380	0.368	0.194	0.015	−0.109	−0.294	0.139	0.351	0.498	0.126
	*p*	0.007 *	<0.001 *	<0.001 *	0.002 *	<0.001 *	0.502	0.064	0.067	0.077	0.365	0.944	0.613	0.163	0.518	0.093	0.013 *	0.556

Abbreviations: Values represent correlation coefficients and corresponding *p*-values. Pearson’s correlation coefficient (r) was used when both variables in the pair were normally distributed, whereas Spearman’s rank correlation coefficient (ρ) was used whenever at least one variable in the pair did not meet the normality assumption. The non-normally distributed variables in the correlation matrix were TD, Acc, Z2, Z5, and PC1; therefore, all correlations involving these variables were calculated using Spearman’s ρ. * = Statistical significance was set at *p* < 0.05. CMJ = countermovement jump; S10 = 10 m sprint; S30 = 30 m sprint; COD = change-of-direction test; VO_2_max = maximal oxygen uptake estimated from the 30–15 Intermittent Fitness Test; TD = total distance; SC = sprint count; SpD = sprint distance; Vmax = maximum velocity; Acc = accelerations; Dec = decelerations; Z1–Z5 = velocity zones; PC1 and PC2 = principal components extracted from principal component analysis. The color scale represents the magnitude and direction of the correlations: light red indicates weak negative correlations, dark red indicates strong negative correlations, light blue indicates weak positive correlations, and dark blue indicates strong positive correlations.

**Table 3:** Factor loadings of physical performance and external load variables across the extracted principal components in professional female football players during the pre-season.

	Component	
	PC1	PC2
CMJ	-	0.537
S10	-	−0.769
S30	-	−0.753
COD	-	−0.602
VO_2_max	-	0.752
TD	0.894	-
SC	0.698	-
SpD	0.798	-
Vmax	0.821	-
Acc	0.773	-
Dec	0.764	-
Z1	0.494	-
Z2	0.811	-
Z3	0.909	-
Z4	0.778	-
Z5	0.573	0.524

Abbreviations: Values represent factor loadings from principal component analysis with varimax rotation. Only loadings ≥ 0.40 were considered for interpretation. PC1 = principal component 1 (predominantly external load variables); PC2 = principal component 2 (predominantly physical performance variables); CMJ = countermovement jump; S10 = 10 m sprint; S30 = 30 m sprint; COD = change-of-direction test; VO_2_max = maximal oxygen uptake; TD = total distance; SC = sprint count; SpD = sprint distance; Vmax = maximum velocity; Acc = accelerations; Dec = decelerations; Z1–Z5 = velocity zones.

## Data Availability

The data presented in this study are available upon reasonable request from the corresponding author. The data are not publicly available due to privacy and ethical restrictions related to the potential identification of the elite athletes included in the study.
